# P05.24. An integrative approach to improving quality of life for underserved women with advanced cancer at the end of life

**DOI:** 10.1186/1472-6882-12-S1-P384

**Published:** 2012-06-12

**Authors:** S Adler, Y Coulter, J Whitford, K Stone, M Acree

**Affiliations:** 1 University of California, San Francisco, San Francisco, USA; 2 Charlotte Maxwell Complementary CLinic, Oakland, USA

## Purpose

The burden of cancer mortality is disproportionately borne by low-income women, but what is known about the experience at end of life (EOL)—including ways to improve quality of life (QOL)—may not apply to underserved women. In order to develop an integrative approach to EOL care that is sensitive to the needs of the underserved, we developed and tested a patient-centered, relationship-based intervention to reduce suffering at EOL by enhancing meaning.

## Methods

This is a before-and-after, mixed method, exploratory study of underserved women with metastatic cancer at EOL. We studied women’s experiences of EOL and tested the feasibility of a narrative QOL intervention known as an “ethical will”—an enduring document that expresses an individual’s values, beliefs, life lessons, hopes, love, and forgiveness as a written legacy. We conducted one-hour, semistructured interviews with patients, as well as their informal caregivers, physicians, and CAM practitioners. Visual-analog-type QOL scales were administered to patients pre/post intervention. Investigators conducted thematic coding of verbatim transcripts using principles of qualitative content analysis and reconciled differences in interpretation through refined definitions and recoding.

## Results

We recruited 55 participants (88% of known eligible) and conducted 111 interviews. Of the 24 visual-analog-type scales, 9 showed a change in the expected direction of 1 point or more and 6 of these were statistically significant despite the very small sample size. Patients reported feeling better physically, having more energy, and feeling more pleasure, while suffering and weariness decreased. Thematic analysis revealed that patients’ concerns over financial needs were not only the most salient issue at EOL, but were also far more pressing than cancer-related health issues.

## Conclusion

Principles of integrative medicine—including addressing mind, body, and spirit—can facilitate the process of decreasing suffering through enhancing meaning. This narrative intervention shows promise for enhancing quality of life at the end of life for underserved women with metastatic cancer.

